# Ascorbic Acid Significantly Decreases Creatine Kinase Plasma Levels in an Animal Model of Statin/Fibrate-Induced Myopathy

**DOI:** 10.1155/2021/5539595

**Published:** 2021-12-29

**Authors:** Mohsen Zabihi, Fatemeh Askarian, Seyedhossein Hekmatimoghaddam, Mohammadreza Rashidi Nooshabadi, Mohammad Sajjad Zabihi, Seyed Ruhollah Mousavinasab

**Affiliations:** ^1^Department of Pharmacology, School of Pharmacy, Shahid Sadoughi University of Medical Sciences, Yazd, Iran; ^2^Yazd Cardiovascular Research Center, Afshar Hospital, Shahid Sadoughi University of Medical Sciences, Yazd, Iran; ^3^Department of Laboratory Sciences, School of Paramedicine, Shahid Sadoughi University of Medical Sciences, Yazd, Iran; ^4^Department of Pharmacology, Faculty of Pharmacy, Ahvaz Jundishapur University of Medical Sciences, Ahvaz, Iran; ^5^School of Medicine, Shahid Sadoughi University of Medical Sciences, Yazd, Iran; ^6^Department of Clinical Pharmacy, Infectious Diseases Research Center, Shahid Sadoughi Hospital, Shahid Sadoughi University of Medical Sciences, Yazd, Iran

## Abstract

**Background:**

Myopathy is one of the side effects of lipid-lowering drugs, especially statins and particularly when combined with a fibrate. To diagnose myopathy and determine its severity, the plasma levels of three enzymes, creatine kinase (CK), aldolase, and lactate dehydrogenase (LDH), are routinely measured. Physical exercise can aggravate the statin-associated muscular disease. The question is whether antioxidants like ascorbic acid (Vit. C) can prevent such myopathy.

**Methods:**

In this experiment, a combination of atorvastatin (ATV, 80 mg/kg/day) and gemfibrozil (GMF, 1000 mg/kg/day) orally for 10 days as well as exercise as forced swimming on days 8, 9, and 10 were used to induce myopathy. Ascorbic acid (50 mg/kg/day, orally) was added to ATV/GMF plus exercise regimen throughout the 10 days in the treatment group. Mean blood levels of CK, aldolase, and LDH were measured in addition to swimming tolerance times.

**Results:**

There was a significantly higher swimming tolerance time (*P* < 0.05) and lower CK levels (*P* < 0.01) in rats receiving ATV/GMF/Vit. C plus exercise compared with rats not taking Vit. C. LDH and aldolase did not decrease significantly.

**Conclusion:**

The results of this study showed that Vit. C can be effective in preventing myopathy caused by fat-lowering drugs.

## 1. Background

Drug-induced myopathy is one of the most common types of muscle disorders, which can be as mild as subtle myalgia with or without slight weakness or as severe as extensive rhabdomyolysis leading to even acute renal failure [1, 2].

The statin group of drugs is mentioned among the frequent causes of myopathies. Regular use of these drugs can significantly prevent or reduce the incidence of atherosclerosis, so these drugs are considered as the first line of treatment for cardiovascular disease caused by hyperlipidemia. Statins act by inhibiting the enzyme 3-hydroxy-3-methylglutaryl-coenzyme A reductase (HMGCAR), while fibrates decrease very low-density lipoprotein and increase high-density lipoprotein. Myonecrosis and rhabdomyolysis due to statins are rare complications and occur in about 0.1% of patients taking these drugs. However, milder forms are more common [3, 4]. Statin intolerance has been defined as unacceptable symptoms and/or laboratory abnormalities prompting their discontinuance, with an increased risk of future atherosclerosis, though [5, 6].

In patients taking statins, the term “statin-associated muscle symptoms” (SAMS) is used to describe the range of complaints. In some patients, autoantibodies to HMGCAR are found which are said to be highly specific for autoimmune myositis or myopathy. Studies have shown that genes such as SLCO1B1 affect the pharmacokinetics and pharmacodynamics of statins (and of course, statin myopathy); the vitamin D receptor gene is involved as well [7, 8].

Some studies suggest that statin-induced myalgia may be due to cellular stress, which is, at least partially, a function of genes involved in the cellular metabolism in skeletal myocytes, as single-nucleotide polymorphisms in the genes mentioned above are more common in patients with statin myalgia [9]. So, it is understandable that antioxidant agents could have beneficial effects on SAMS. The susceptibility to myopathy is greater in patients receiving concurrent therapy with a number of drugs, particularly those that inhibit CYP3A4 [10], as well as with fibrates [11].

Measurement of plasma level of some enzymes which have a high intracellular concentration in striated muscle cells and are released into the circulation during muscle injury is the standard practice for diagnosis, follow-up, and monitoring of improvement of muscle damage in patients with muscle complications. Creatine kinase (CK) is the most widely used enzyme in this regard. It is more specific to skeletal muscle, rapidly appears in blood after a muscular injury, and can be determined conveniently by simple photometric reactions in most clinical laboratories. Physical activity may increase serum CK levels. Aldolase is found in most body tissues but mainly in the skeletal muscle, liver, and brain. It is an enzyme involved in the glycolytic pathway. Although an increase in aldolase levels is not as sensitive and specific as CK levels for myopathy diagnosis, it is sometimes increased in patients with myositis who have normal CK levels [12]. Another valuable enzyme for diagnosing and monitoring muscular disorders is lactate dehydrogenase (LDH), which converts pyruvate to lactate. Its drawback is that many tissues other than muscles have high concentrations of LDH, which are released upon cell membrane dysfunction [13].

Ascorbic acid is an essential water-soluble vitamin that acts as a cofactor and antioxidant. It is necessary for collagen hydroxylation, carnitine biosynthesis, and hormone/amino acid production [14].

The present study aimed to evaluate the effect of ascorbic acid on the improvement of statin/fibrate-induced myopathy in rats. Our previous studies (unpublished data) showed that a combination of atorvastatin (ATV) and gemfibrozil (GMF) plus exercise (forced swimming test) is a reliable model for induction of myopathy.

## 2. Methods

### 2.1. Chemicals

The drugs used in this study included ascorbic acid and gemfibrozil (both from Darupakhsh Co., Iran) as well as atorvastatin calcium (Sobhan Co., Iran), which were dissolved in distilled water to prepare solutions for feeding rats by tube gavage every morning at 9 am.

### 2.2. Animals

This study was performed on 30 male Wistar rats, approximately 56 days old and weighing 250–300 g, which were kept in the animal house of the Faculty of Pharmacy, Shahid Sadoughi University of Medical Sciences, Yazd, Iran. They were kept in standard cages (*n* = 6 in each cage) with usual temperature and humidity control (20–25°C, 50–60% relative humidity) and 12 h light/dark cycles. The rats had free access to tap water and standard food. This animal study received an ethics code (IR.SSU.MEDICINE.REC.1398.183) by the Shahid Sadoughi University of Medical Sciences, Yazd, Iran, regarding care and work on laboratory animals. Rats were randomly allocated to five groups (*n* = 6): a control group that took vehicle (distilled water) without any swimming; a control plus swim group that received the vehicle and underwent forced swimming test on days 8, 9, and 10; an ATV/GMF without swim group which was fed gavage ATV (80 mg/kg/day) and GMF (1000 mg/kg/day) for 10 days without swimming; an ATV/GMF plus swim group that received the same ATV and GMF doses for 10 days and had forced swim on days 8, 9, and 10; and an ATV/GMF/Vit. C plus swim group which had the same doses of ATV and GMF but were also treated with 50 mg/kg/day of ascorbic acid orally for 10 days in addition to forced swimming on days 8, 9, and 10. The doses were based on the results of our previous study (submitted to a peer-reviewed journal but not published as yet). The weights of the rats were measured on day 10 using an electronic balance. At the end of the study, the rats were sacrificed under deep anesthesia with ketamine.

### 2.3. Forced Swimming Test

For maximum physical activity, a previously published method [15] was modified, so that the animals were placed separately in a large glass cylinder containing water (25°C). From the beginning of swimming to near-drowning due to fatigue, the duration of their movements was recorded by the chronometer. The average time in three days was considered as swimming tolerance (i.e., endurance) time.

### 2.4. Plasma Enzyme Activity Measurements

On the 10th day of the study, the rats were given deep anesthesia using ketamine (50 mg/kg) and xylazine (10 mg/kg), and 3 mL of blood was drawn from the heart and put in plastic tubes containing K2-EDTA. The plasma was sent to the laboratory (Central Laboratory, Yazd, Iran) for quantitative measurement of the enzyme levels according to instructions of approved biochemical UV-spectrophotometric assay kits, all employing kinetic reactions at 37°C. The CK-NAC-LQ kit (Audit Diagnostics, Ireland) was used for determining the CK level. Measurement of aldolase was similar by employing a commercial kit (Biorexfars, Iran). LDH measurement was performed using a kit produced by Bionik, Iran. The stated linearity ranges for measuring CK, aldolase, and LDH were 2–2000 U/L, 1–28 U/L, and 2–1450 U/L, respectively.

### 2.5. Statistical Analysis

To compare the mean of plasma enzyme levels, swimming tolerance times, and weights of animals in the five groups, one-way analysis of variance (ANOVA) was used. Tukey' post hoc test was used to compare multiple groups. A statistically significant difference was defined as a *P* value less than 0.05.

## 3. Results

### 3.1. Weights of the Rats

The mean weights of each group at the end of the study were not significantly different (*P* value >0.05).

### 3.2. Swimming Tolerance Time

**Figure 1:**
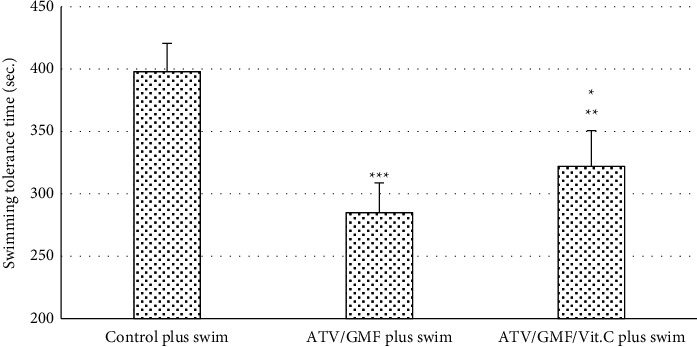
Mean of the swimming tolerance times of rats in 3 groups. ATV, atorvastatin (80 mg/kg/day for 10 days); GMF, gemfibrozil (1000 mg/kg/day for 10 days); Vit. C, ascorbic acid (50 mg/kg/day). Swimming was done in days 8, 9, and 10. The levels of each bar are mean, and the error bar is SEM (*n* = 6 in each group). ^∗∗∗^*P* value <0.001 and ^∗∗^*P* value <0.01 in comparison with the control group. ^∗^*P* value <0.05 in comparison with the ATV/GMF plus swim group. One-way ANOVA followed by Tukey' post hoc test.

The mean of the swimming tolerance times at days 8, 9, and 10 were calculated in the three groups which had forced swimming. It showed a statistically significant difference (*P* value <0.001) between the control plus swim group and ATV/GMF plus swim group. Vit. C increased swimming tolerance time in the ATV/GMF/Vit. C group vs. ATV/GMF (*P* < 0.05), while it was significantly less than the control group (*P* < 0.01). Compared with the control plus swim group, there was nearly 30% reduction in the swimming tolerance time in the ATV/GMF plus swim group. However, in the ATV/GMF/Vit. C plus swim group, the reduction in the swimming tolerance time was about 20% (Figure 1).

### 3.3. Levels of CK

**Figure 2:**
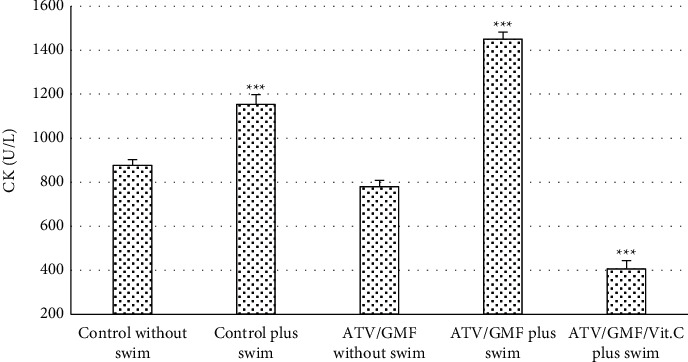
Plasma creatine kinase (CK) levels of rats in the control and treated groups. ATV, atorvastatin (80 mg/kg/day for 10 days); GMF, gemfibrozil (1000 mg/kg/day for 10 days); Vit. C, ascorbic acid (50 mg/kg/day for 10 days). Swimming was done in days 8, 9, and 10. The levels of each bar are mean, and the error bar is SEM (*n* = 6 in each group). ^∗∗∗^*P* value <0.001 compared with all of the other groups. One-way ANOVA followed by Tukey' post hoc test.

Levels of the enzyme CK in plasma were significantly higher in the control plus swim group and ATV/GMF plus swim group than in other groups (*P* value <0.001). The ATV/GMF/Vit. C plus swim group had significantly lower CK levels than all of the other groups (*P* value <0.001). The CK levels in the ATV/GMF without swim group and control without swim group were not significantly different (Figure 2).

### 3.4. Levels of LDH

**Figure 3:**
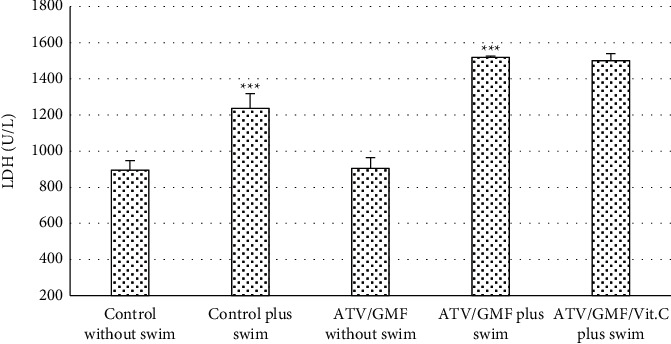
Comparison between control and treated groups of rats regarding plasma lactate dehydrogenase (LDH) levels. ATV, atorvastatin (80 mg/kg/day for 10 days); GMF, gemfibrozil (1000 mg/kg/day for 10 days); Vit. C, ascorbic acid (50 mg/kg/day for 10 days). Swimming was done in days 8, 9, and 10. The levels of each bar are mean, and the error bar is SEM (*n* = 6 in each group). ^∗∗∗^*P* value <0.001 in comparison with the groups which did not have swimming. LDH levels did not decrease significantly in the ATV/GMF/Vit. C plus swim group compared with the ATV/GMF plus swim group (*P* value >0.05). One-way ANOVA followed by Tukey' post hoc test.

Plasma levels of LDH happened to be significantly more in the control plus swim group and ATV/GMF plus swim group vs. the other groups which did not swim (*P* value <0.001). However, the LDH levels of the ATV/GMF without swim group were not significantly different from the control without swim group. LDH levels did not decrease significantly in the ATV/GMF/Vit. C plus swim group in comparison with the ATV/GMF plus swim group (*P* value >0.05) (Figure 3).

### 3.5. Levels of Aldolase

**Figure 4:**
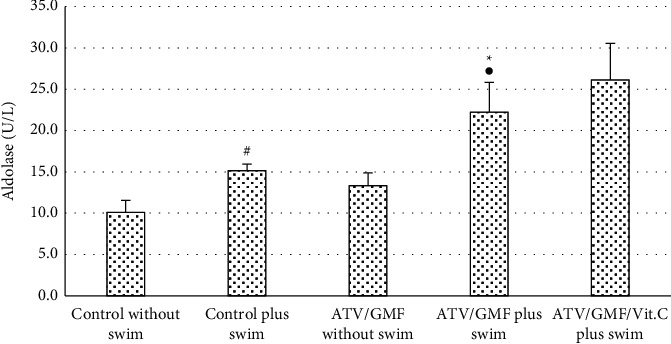
Aldolase levels of rats were compared between control and treated groups. ATV, atorvastatin (80 mg/kg/day for 10 days); GMF, gemfibrozil (1000 mg/kg/day for 10 days). Swimming was done in days 8, 9, and 10. The levels of each bar are mean, and the error bar is SEM (*n* = 6 in each group). ^∗^*P* value <0.01 compared with the control without swim group. *P* value <0.05 in comparison with the ATV/GMF without swim group. ^#^*P* value <0.05 in comparison with the control without swim group. One-way ANOVA followed by Tukey' post hoc test.

Plasma aldolase activity was significantly higher in the control plus swim group vs. the control without swim group (*P* value <0.05). Plasma aldolase levels of the ATV/GMF plus swim group were also significantly more than the control without swim group (*P* value <0.01) and again significantly above the levels in the ATV/GMF without swim group (*P* value <0.05). Plasma aldolase levels in ATV/GMG/Vit. C group plus swim were a little more than the ATV/GMF plus swim group but not significantly (*P* value >0.05) (Figure 4).

## 4. Discussion

A common side effect of statins is myopathy, and this complication can be exacerbated by the concomitant use of some other drugs, especially those that inhibit CYP3A4 as the cytoplasmic enzyme responsible for the metabolization of statins such as simvastatin, lovastatin, and atorvastatin [10]. A similar effect is also seen when statins are coprescribed with fibrates [16]. Muscle toxicity with fibrates has also been reported and is more common in patients taking statins simultaneously. The mechanism is under debate. Glucuronidation, an essential pathway for renal excretion of lipophilic statins, appears to be significantly inhibited by gemfibrozil [17].

The incidence of myopathy in combined administration of some statins and gemfibrozil is estimated at about 1–5% [18, 19], and rhabdomyolysis is the most severe complication observed [4]. By changing the type of fibrate (for example, fenofibrate poses the least risk) and the use of statins in relatively low doses, muscle toxicity can be reduced because the adverse effects are dose-dependent [20, 21]. Concomitant administration of statins and fibrates is common in patients with hypertriglyceridemia and hypercholesterolemia, which prompted us to design the present study.

Physical activity intensifies statin-associated muscle symptoms (SAMS) [22] and leads to increased plasma CK levels, which are more visible in the uninitiated. Therefore, if a gradual increase in exercise is followed in these people and sufficient time is provided for adaptation and metabolic clearance of drugs, the risk of myopathy will be lower. However, even without such adaptation, exercise-induced muscle damage is usually mild and subclinical [22].

Our project exposed the rats to a sudden challenge of forced maximal exercise to see the highest possible muscle injury. Hence, the serum levels of CK, aldolase, and LDH were significantly higher in rats with forced physical activity than those without this challenge. The rats receiving combined ATV and GMF showed a further increase in the plasma enzyme levels. Swimming tolerance time was strikingly decreased (*P* value <0.001) in rats consuming ATV and GMF, supporting the synergistic effect of exertion and drug.

A few other studies in this field are available to compare our work with. Ozaki et al. developed a model for statin-induced myopathy using skeletal muscle HMGCAR knockout mice. Results of that study showed postnatal myopathy with high serum CK levels and myonecrosis, which underlines the role of HMGCAR in the metabolization of statins [23]. In another work, Nakahara et al. induced myopathy by HMGCAR inhibitors in rabbits, followed by histopathological examination of skeletal muscle and measurement of plasma CK. That study revealed light microscopic muscle fiber necrosis and degeneration, altered acid phosphatase activity in cells, and electron microscopic alterations including autophagic vacuoles, swelling of mitochondria, disruption, and hypercontraction of myofibrils [24].

In the current research, the most dramatic effect of ascorbic acid was the prevention of CK rise, the primary biomarker of skeletal muscle damage [25], in the ATV/GMF plus swim group. It increased swimming tolerance time in the drug-induced myopathy group too. Ascorbic acid could not alter elevated LDH or aldolase levels in the drug-induced myopathy group, though. Perhaps the molecular mechanism is that ascorbic acid provides electrons needed for reducing oxygen; the antioxidant capabilities are also shared by several other compounds, including vitamin E and folic acid. It is also a cofactor for the reduction of folate to dihydro and tetrahydrofolate [14].

Ascorbic acid is involved in the following biologic processes. (1) Fatty acid transport: the transport of long-chain fatty acids across the mitochondrial membrane is a carnitine-dependent process, and carnitine synthesis requires ascorbic acid as an electron donor [26]. (2) Collagen synthesis: formation of collagen requires enzymatic hydroxylation of two amino acids, proline and lysine; ascorbic acid is an electron donor in reactions catalyzed by the enzymes prolyl hydroxylase and lysyl hydroxylase, which form hydroxyproline and hydroxylysine, respectively. Failure in collagen synthesis results in impaired wound healing, defective tooth formation, and deficient osteoblast and fibroblast function. (3) Synthesis of the neurotransmitter norepinephrine involves hydroxylation of dopamine by the enzyme dopamine beta-mono-oxygenase, where ascorbic acid is a required cofactor. (4) Metabolism of prostaglandin and prostacyclin: it may be capable of attenuating the inflammatory response or even sepsis syndrome [24]. (5) Nitric oxide synthesis: ascorbic acid may promote nitric oxide synthesis, a potent vasodilator [27, 28]. (6) Mitochondrial health: regarding the extreme dependence of muscle activity to mitochondria for ATP, it is clear that mitochondrial injuries in general result in oxidative stress, i.e., higher levels of reactive oxygen species [29, 30] leading to cell membrane damage through lipid peroxidation.

## 5. Conclusion

Ascorbic acid decreases CK levels in statin/fibrate-induced myopathy and improves rats' tolerance to skeletal muscle activity. Ascorbic acid may be promising in reducing muscle injury induced by lipid-lowering medications.

## Data Availability

The datasets used and/or analyzed during the current study are available from the corresponding author upon request.

## Abbreviations

ANOVA: Analysis of variance
CK: Creatine kinase
LDH: Lactate dehydrogenase
Vit. C: Vitamin C
ATV: Atorvastatin
GMF: Gemfibrozil
SAMS: Statin-associated muscle symptoms.

## Supplementary Materials

Supplementary Materials The graphic design of the research methodology is shown in the supplementary file.
