# Supplementary material for: Ascorbic Acid Significantly Decreases Creatine Kinase Plasma Levels in an Animal Model of Statin/Fibrate-Induced Myopathy
Source: Adv Pharmacol Pharm Sci. 2021 Dec 29;2021:5539595. doi: 10.1155/2021/5539595 (PMC8731278; doi:10.1155/2021/5539595)
Supplement: Supplementary Materials — The graphic design of the research methodology is shown in the supplementary file. [file 5539595.f1.docx]

30 male Wistar rats, 250-300 g, randomly divided

Control, water

Water+swim

ATV+GMF

ATV+GMF+

swim

ATV+GMF+

swim+Vit. C

ATV: atorvastatin, 80 mg/kg/day, gavage for 10 days;

GMF: gemfibrozil, 1000 mg/kg/day, gavage for 10 days;

Vit. C: vitamin C, 50 mg/kg/day, gavage for 10 days;

Swim: forced swimming on days 8, 9 and 10

Weighing on the day 10, sacrifice, drawing blood from the heart

Determination of plasma creatine kinase, aldolase and lactate dehydrogenase levels
